# Bullying victimization among middle school students in el kef governorate (Tunisia)

**DOI:** 10.1093/eurpub/ckac131.497

**Published:** 2022-10-25

**Authors:** A Amara, A Mtiraoui, D Nammouchi, J Sahli, M Mellouli, A Mtiraoui, C Zeddini, M El Ghardellou

**Affiliations:** 1 Department of Family and Community Medicine, Faculty of Medicine of Sousse, Sousse, Tunisia; 2 Research Laboratory, LR12ES03, Sousse, Tunisia; 3 Department of Psychiatry, Farhat Hached University Hospital, Sousse, Tunisia

## Abstract

**Background and aim:**

Bullying is a complex and widespread public health issue that one can be exposed to at any age and at any field, but it is considered particularly frequent during times of transition in children’s and adolescents’ lives. This study aims to investigate the prevalence and various forms of bullying victimization and explore culture of bullying victimization among students.

**Methods:**

A cross-sectional study was conducted during the Academic year 2018/2019 among middle school students in the governorate of El Kef, Tunisia. The sampling method adopted for this research was a cluster sampling technique. Data were collected using self-administered questionnaires. The Students’ involvement in bullying victimization was assessed using a validated Arabic version of the revised Olweus Bully/Victim Questionnaire.

**Results:**

A total of 1111 middle school students were enrolled. The prevalence of bullying victimization behavior in this study was 45.8 % [95% CI: 45.5- 46]. Looking at the forms of bullying experienced by students: “Being called mean names” was the most prevalent form of being bullied with 26.9% (n = 299), followed by 16.9% reported “being kicked in some place”, 16.3% reported “being bullied through false rumors” and 14.3% experienced bullying through messages, calls or images by means of mobile phones or Internet and 8.1% of the students reported being bullied with sexual gestures or comments. Verbal bullying (29.5%) was the most common type of victimization, followed by physical victimization, relational victimization and cyber victimization with 22.5%, 22.2% and 14.3% respectively. More than half of the students (58.5%) were more likely to inform others about incidents of bullying in their schools, mainly their parents (36.6%) or a friend (32.6%).

**Conclusions:**

Bullying is serious and major public health issue that have a negative impact on adolescents’ well-being, and require special attention at the family, school, and community level

**Key messages:**

• High prevalence of bullying victimization with predominace of verbal type.

• Culture of bullying victimization and taking care of victims is still weak.

